# Screening and characterization of *in-vitro* probiotic criteria of *Saccharomyces* and *Kluyveromyces* strains

**Published:** 2018-04

**Authors:** Reyhaneh Moradi, Rahim Nosrati, Hamed Zare, Tahereh Tahmasebi, Horieh Saderi, Parviz Owlia

**Affiliations:** 1 Department of Microbiology, Faculty of Advanced Sciences & Technology, Pharmaceutical Sciences Branch, Islamic Azad University, Tehran, Iran; 2 Department of Pharmaceutical Biotechnology, School of Pharmacy, Mashhad University of Medical Sciences, Mashhad, Iran; 3 Department of Pharmaceutical Biotechnology, School of Pharmacy, Shahid Beheshti University of Medical Sciences, Tehran, Iran; 4 Molecular Microbiology Research Center (MMRC), Faculty of Medicine, Shahed University, Tehran, Iran

**Keywords:** Probiotic, *Saccharomyces*, Yeast, *Kluyveromyces*

## Abstract

**Background and Objectives:**

Probiotics are defined as live micro-organisms conferring a health benefit on the host. Although most probiotics are bacteria, some yeasts such as *Saccharomyces* and *Kluyveromyces*, has been found to have effective probiotic properties. The objective of this study was to isolate and identify indigenous *Saccharomyces* and *Kluyveromyces* yeast strains and to compare some probiotic characteristics between these two strains *in vitro*.

**Materials and Methods:**

Strains were isolated on yeast glucose chloramphenicol agar medium from 205 samples and identified by morphological, physiological and biochemical assays. The effects of different conditions such as pH and temperature on the survival and growth of the isolates were studied. In addition, resistance to acidic pH (1.5, 2, 3 and 5), pepsin and different concentrations of bile salts (1%, 3% and 5%), as well as proteolytic, lipolytic and hemolytic activity of selected isolates were assessed. Finally, the best isolates were selected for investigation of their viability in samples of dairy products.

**Results:**

126 isolates were identified using biochemical and molecular techniques as yeast strains. Five isolates were found to have effective probiotic properties, belonging to *Kluyveromyces marxianus* (S97, S101 and S106) and *Saccharomyces cerevisiae* (S28, S34). These isolates were able to grow at 37°C, pH=1.5, withstand to concentration of 5% oxbile and pepsin and exhibit the proteolytic activity. The isolates of *K. marxianus* showed better viability in dairy (yogurt).

**Conclusion:**

In the *in-vitro* comparative experiments, the isolates of *K. marxianus* showed better probiotic potentials.

## INTRODUCTION

Nowadays, much attention has been devoted to the development of useful foods that have probiotic microbial strains responsible for health-promoting effects. Probiotics are defined as: “viable microorganisms which, when administered in adequate amounts, confer a beneficial effect on the health of the host” (1). To improve human health, probiotic microorganisms have been added into food as dietary adjuncts. Dairy products such as yogurt, cheese and fermented milk are mostly used as probiotic sources for transporting probiotic microorganisms to the human gastrointestinal tract (2–4). Although lactic-acid bacteria and bifidobacteria are predominantly probiotic microorganisms (5), some yeasts such as *Saccharomyces* and *Kluyveromyces* isolates are also considered as probiotic due to their effective properties (6, 7).

*Saccharomyces* is yeast which is most commonly used for animal and human consumption in the food industry worldwide. For long times, *Saccharomyces cerevisiae* var. boulardii (*Saccharomyces boulardii*) has been the only yeast available in various commercial formulations as a probiotic for humans (8, 9). Numerous beneficial effects of *S. boulardii* have been demonstrated including the modulation of immune response, decreasing inflammation, regulation of the intestinal flora, treatment of diarrhea caused by bacteria and several prophylactic and therapeutic effects in inflammatory gastrointestinal diseases (for a review see Kelesidis and Pothoulakis (10).

Investigations for other yeasts with probiotic potential have resulted in finding *Kluyveromyces marxianus fragilis* B0399 which is the first non-*Saccharomyces* yeast approved as a probiotic for animal feeding and human consumption (11). This strain has been recommended as biological agent added to food and feed in the European Food Safety Authority list (12). *K. marxianus* is a lactic yeast which is isolated from different dairy products such as whey, curds of cow milk, kefir and cheese (2, 13). However, little is known about the positive impact of *K. marxianus* clinical and experimental evidences has demonstrated its role in gastrointestinal diseases (2). *K. marxianus* is known to modulate the immune response and decrease the level of pro-inflammatory cytokines (14, 15). Also, this yeast adheres to the enterocytes of the intestinal epithelium (15) and regulates intestinal activity during antibiotic therapy (16). Moreover, due to its β-galactosidase activity which is absent in strains of the genus *Saccharomyces, K. marxianus* is able to reduce the lactose content; this characteristic help susceptible individuals benefit from lowering the effects of lactose intolerance (17).

The aim of the present study was to isolate and characterize the probiotic criteria of the food-related *Kluyveromyces* and *Saccharomyces* yeasts including the tolerance to gastric acidity and bile salts as well as proteolytic, lipolytic and hemolytic activity. The viability of these strains during of refrigerated storage and shelf-life in the dairy product (yogurt) was evaluated as well.

## MATERIALS AND METHODS

### Yeast isolation and identification.

Two hundred-five sweet fruit and dairy samples were collected from April 2014 to March 2015. One gram of each sample was added into 250 ml Erlenmeyer flask containing 90 ml of normal saline, serial dilutions were prepared in peptone water (0.1%) up to 10^−6^. 500 μl of the dilutions 10^−4^, 10^−5^ and 10^−6^ were put into plates containing Sabouraud dextrose agar. Yeast strains were isolated by surface-streaking onto yeast-extract glucose chloramphenicol (YGC) agar medium and incubated at 30°C for 2–5 days in aerobic conditions (6, 18). The pure cultures of isolates were preserved on broth sabouraud by freezing at −70°C with 15% (v/v) of glycerol for longer preservation. Yeasts were characterized by microscopic morphology and biochemical characteristics, including growth in malt extract, growth at 37°C, and the ability to hydrolyze urea and ferment sugar (glucose, sucrose, maltose, galactose and lactose) (19, 20). Yeasts were then identified according to the criteria of Kurtzman et al. (20).

### PCR amplification and sequencing analysis.

The yeasts were also characterized by molecular techniques based on the amplification and sequencing of the ribosomal DNA internal transcribed spacer region (ITS) and D1/D2 domain of 26S rDNA. The primers used to amplify the ITS/5.8S rDNA were ITS1 (5′-CGG GAT CCG TAG GTG AAC CTG CGG-3′) and ITS4 (5′-CGGGAT CCT CCG CTT ATT GAT ATG C-3′) (6). The D1/D2 variable domains were amplified using the primers NL-1 (5′-GCATATCAATAAGCGGAGGAAAAG-3′) and NL-4 (5′-GGTCCGTGTTTCAAGACGG-3′) (21). The purified products were sent to Iranian Biological Research Center (Tehran, IRAN) for DNA sequencing. The obtained sequences were compared with those included in the GenBank database using the Basic Local Alignment Search Tool (BLAST at http://www.ncbi.nlm.nih.gov) (22).

### *In vitro* selection of the probiotic candidates: Survival at different temperatures.

The effect of temperature on the growth of strains was examined by adding 100 μl of the active yeast suspensions (∼10^9^ CFU/ml of each isolate) into sabouraud dextrose broth and incubation at 25, 30, 37 and 42°C.

### Bile tolerance.

100 μl of the active yeast suspensions (∼10^9^ CFU/ml of each isolate) was added into sabouraud dextrose broth with different concentrations (1%, 3% and 5% w/v) of oxgall. The bile tolerance of strains was evaluated based on estimating the number of viable cells after 0, 24, 48, 72 and 96 h incubation at 37°C in triplicates.

### Survival at low pH.

The growth at acidic pH was evaluated by inoculating of 100 μl of activated strains suspensions (∼10^9^ CFU/ml) into sabouraud dextrose broth by initial pH of 1.5, 2.0, 3.0 and 5.0. The media were incubated at 37°C and the number of viable cells was determined after 0, 24, 48, 72 and 96 h incubation in triplicates.

### Survival in gastric juice.

To determine the viability of strains in the presence of pepsin, simulated gastric juice was prepared by suspending 3 mg/mL pepsin in sterile NaCl solution (0.9% w/v) and adjusted by HCl to achieve pH 2.3 and was inoculated with 20 μl active yeast cultures (∼10^9^ CFU/ml of each isolate). After 90 min of incubation at 37°C, 10 μl of the suspension was added to Sabouraud dextrose broth media and incubated at 37°C. Then, the viable yeast cells were enumerated on Sabouraud dextrose agar plates after 0, 24, 48, 72 and 96 h of incubation in triplicates.

### Proteolytic activity.

To determine the proteolytic activity of strains, 20 μl of selected isolates suspensions (∼10^9^ CFU/ml) were spotted on skim milk agar medium. The inoculated plates were incubated at 37°C for 72 hours. Colonies having a clearance (halo) surrounding them were considered as strains with proteolytic activity (23).

### Lipolytic activity.

For lipolytic activities of strains, 20 μl of selected isolates suspensions (∼10^9^ CFU/ml) were spotted on egg yolk agar medium. The inoculated plates were incubated at 37°C for 72 h. The appearance of an areola around the colonies indicated lipolytic activity of strains.

### Hemolytic activity.

The hemolytic activity assay was carried to evaluate the pathogenicity of isolates which could serve as the criteria for choosing a probiotic strain. The strains were tested for hemolytic activity using blood agar (7% v/v sheep blood) as described by Foulquie Moreno (24). Briefly, 20μl of selected isolates suspensions (∼10^9^ CFU/ml) was spotted onto sterile blood agar. Plates were incubated at 37°C for 48 h and then were observed for lyses zones around the colonies (positive reaction or β-hemolysis). The non-hemolytic reaction was recorded by observation of green-hued zones around the colonies (α-hemolysis) or did not produce any effect on the blood plates (γ-hemolysis) (25). A strain of *S. aureus* was used as positive control.

### Inoculation of dairy products.

Growth and survival experiments were conducted in two different batches of 500 ml low-fat yogurt (Kalleh dairy company-Iran). The yoghurt samples were bought from a superstore. Samples were transported in a cool box on ice and keep cold upon arrival in the laboratory. All the dairy products were aseptically dispensed at the same amount (100 ml) into sterilized Schott bottles. Samples were inoculated within 12 h of reception at approximately 1 ml of selected isolates suspensions (∼10^9^ CFU/ml) to achieve an initial yeast count of > 10^6^ CFU/ml (> 7 log10 CFU/ml) in the dairy products. The products were regularly mixed. The inoculated yogurts were stored at 4°C and 25°C for 14 days. A non-inoculated yogurt was used as control. Every 2 days, the number of viable cells was determined and the growth curves were drawn and simultaneously, the pH of each sample was monitored (26).

Data analysis and graph drawing were done using statistics software Graph Pad Prism (v5.0.4) and the Microsoft Excel program.

## RESULTS

From 205 samples, a total of 126 isolates, named S1 to S126, were isolated and initially identified as yeast strains by means of classical microbiological and biochemical tests following the criteria of Kurtzman and Fell (20). In a series of primary comparative experiments in plate assays, 32 strains showed survival after exposure to pH 2.0 and in presence of 3% (w/v) bile salts and were therefore selected for further characterization. Among these strains, we identified 5 strains belonging to species *Saccharomyces cerevisiae* (2 strains; S28 and S34), and *K. marxianus* (3 strains; S97, S101 and S106). The sequences data revealed that S28 and S34 strains are *S. cerevisiae* (GenBank accession Nos. CBS 8274 and CBS 6414) and S97, S101 and S106 strains are *K. marxianus* (GenBank accession Nos. CBS 5673, JX174415 and CBS 712, respectively). Subsequent studies were performed on comparison of probiotic potential of these strains.

**Fig. 1:**
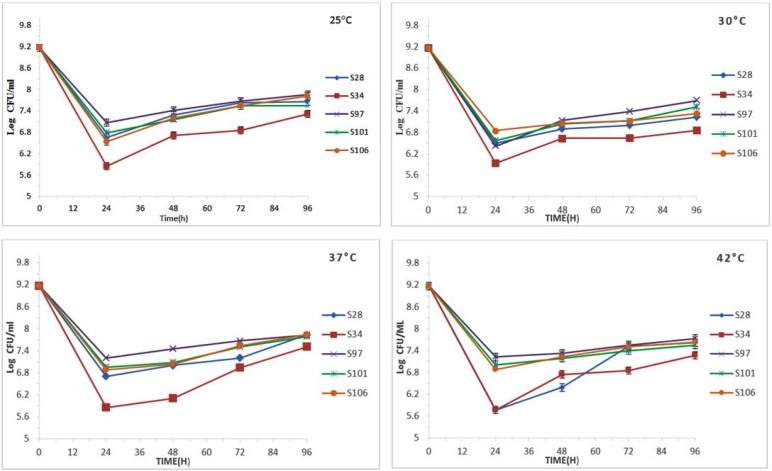
The effect of different incubation temperature (25, 30, 37 and 42°C) on growth of the selected isolates of *Kluyveromyces marxianus* (S97, S101 and S106) and *Saccharomyces cerevisiae* (S28 and S34). All data points are the means of three replicates. Standard errors are shown by vertical bars.

Generally, all of isolates are able to grow at temperatures of 25–42°C. However, the growth of yeasts was reduced at 42°C (Fig. 1). The minimum viability levels of all isolates were reached after 24 h of incubation. *K. marxianus* S97 showed the highest growth level (7.83, based on Log CFU/ml) during 96 h, whereas *S. cerevisiae* S34 strain had the lowest cell survival (7.51) during the same period of time when incubated at 37°C (physiological temperature). At temperature above body temperature (42°C), survival rate of *Kluyveromyces* strains (S97, S101 and S106) was higher than strains of *Saccharomyces* (S28 and S34).

**Fig. 2:**
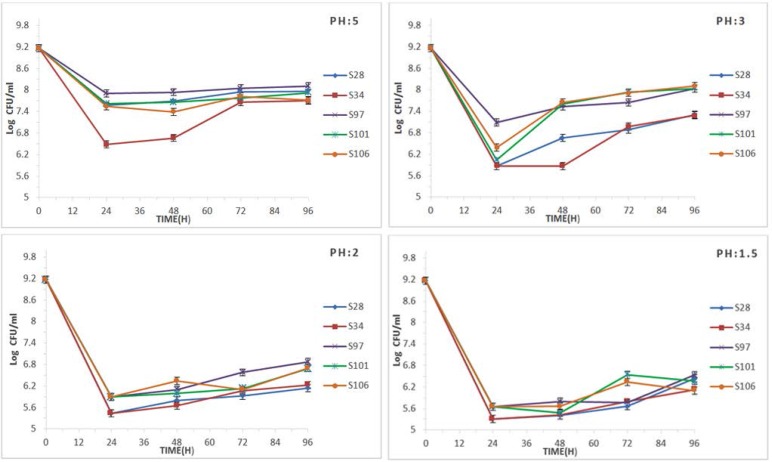
Ability of the selected isolates *Kluyveromyces marxianus* (S97, S101, and S106) and *Saccharomyces cerevisiae* (S28 and S34) to tolerate low pH. All data points are the means of three replicates. Standard errors are shown by vertical bars.

The selected yeasts were able to grow under stressful environments, showing resistance to acid pH and high concentrations of bile salts. With respect to the effect of oxgall concentration, all the 5 selected yeasts were able to grow even when the bile salts concentration was as high as 5 % (w/v); however, increased bile salt concentration in medium was associated with a gradual decrease in the rate of growth (Fig. 2). Under such conditions, as shown in Fig. 2, the maximum growth rate of isolates were obtained for *K. marxianus* S101 and *S. cerevisiae* S28 strains. Therefore, these isolates were exhibited strong resistance to bile salts. In comparison, *S. cerevisiae* S34 was the most sensitive strain to bile salts.

**Fig. 3:**
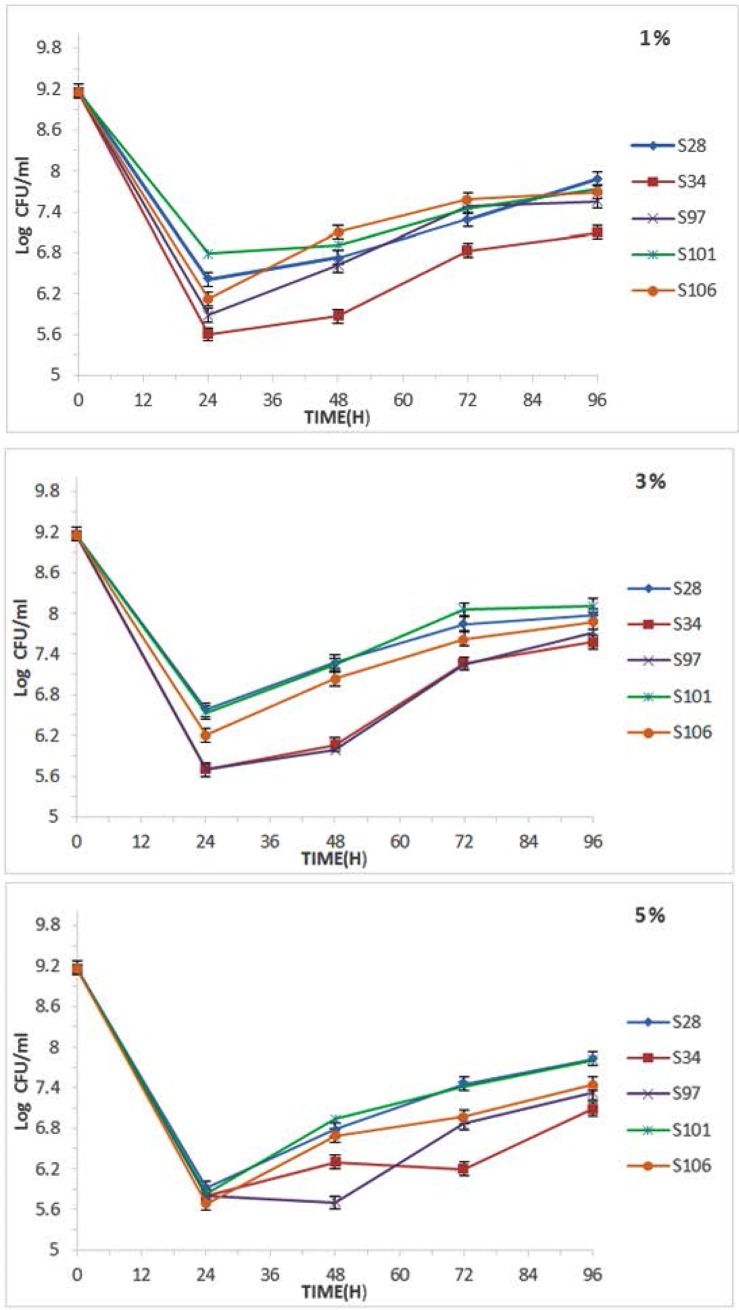
Survival and growth of the selected isolates *Kluyveromyces marxianus* (S97, S101 and S106) and *Saccharomyces cerevisiae* (S28 and S34) under high concentrations of bile salts. All data points are the means of three replicates. Standard errors are shown by vertical bars.

In this work, medium inoculation at various initial acidic pH values (1.5, 2.0, 3.0 and 5.0) resulted in survival of all the strains at pH 3.0 and 5.0 after 96 h incubation (Fig. 3). However, after incubation in low pH (1.5 and 2.0), cell counts of all strains were reduced. In other words, the least amount of growth was detected in pH 1.5. At pH value 1.5, the highest viability (based on Log CFU/ml) was 6.53 for *K. marxianus* S97. *S. cerevisiae* S34 showed the lowest viability (the number of yeast cells) at various initial pH values. As shown in Fig. 3, strains of *Kluyveromyces* (S97, S101 and S106) showed a survival rate higher than strains of *Saccharomyces* (S28 and S34) when pH was decreased.

**Fig. 4:**
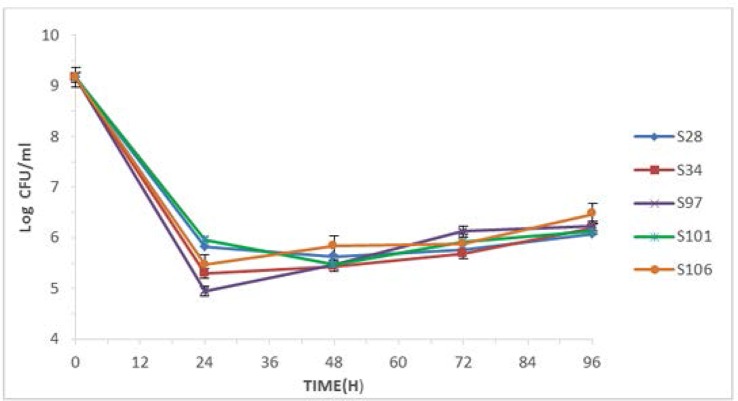
Resistance of the selected *Kluyveromyces marxianus* strains (S97, S101 and S106) and *Saccharomyces cerevisiae* strains (S28 and S34) to simulated gastrointestinal conditions. All data points are the means of three replicates. Standard errors are shown by vertical bars.

The potential ability of the 5 mentioend strains to withstand during the passage through the human gastrointestinal tract as assayed indirectly *in vitro* is demonstrated in Fig. 4. Under the conditions used in this study, strains had a similar survival patterns and were only slightly affected by exposure to the gastric juice. The number of surviving cells increased in the presence of pepsin. Strains of *Kluyveromyces* (S97 and S106) exhibited strong resistance to gastric juice (Fig. 4). The results also showed that these isolates exhibited proteolytic activity while lipolytic and haemolytic tests were negative for each selected isolate.

**Fig. 5:**
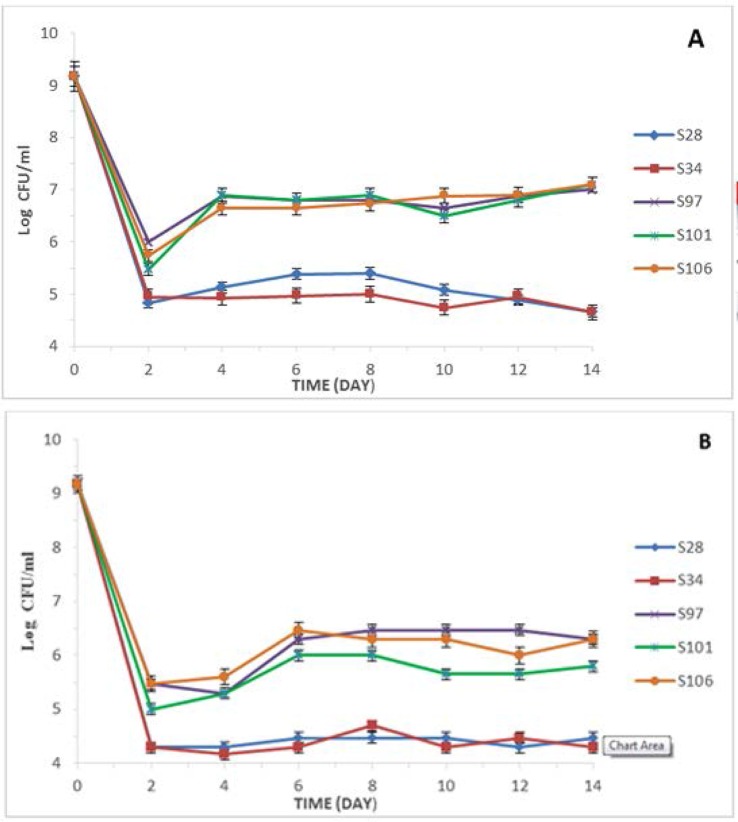
Growth and viability of the selected yeast *Kluyveromyces marxianus* strains (S97, S101 and S106) and *Saccharomyces cerevisiae* strains, S28, S34, in dairy products (yoghurt) at 5°C (panel A) and 25°C (panel B). All data points are the means of three replicates. Standard errors are shown by vertical bars.

In a comparative experiment set up for the viability of strains at the storage period, the results of inoculation of dairy products showed that, in all treatments, the initial yeast count (8.6 based on Log CFU/ml) was decreased in the first two days. Fig. 5 demonstrates that as incubation was extending for more than 2 days, the growth rate for Kluyveromyces isolates was gradually improved at both 4°C (Fig. 5A) and 25°C (Fig. 5B), so that the cell population of these strains survived and increased slightly from 4.7 log10 CFU/ml to 7.1 log10 CFU/ml over the storage period at 4°C. In contrast, a different pattern was observed for *Saccharomyces* strains. The number of strains of *Saccharomyces* S28 and S34 extended beyond 2 days of inoculation was remained practically stable. The highest count of yeasts was evidenced at 4°C for *Kluyveromyces* isolates S97, S101 and S106 (7.1 Log10 CFU/ml) whereas, under same conditions, the lowest levels of growth were detected for Saccharomyces strains (4.65 based on Log CFU/ml). Fig. 5 indicates that the viability of *Saccharomyces* strains in yogurt samples was lower than *Kluyveromyces* isolates at both high and low temperature. The pH of the different yeast inoculated dairy products changed from 0.1 to 0.5 over the storage period. No yeasts were detected in any of the control samples (data not shown).

## DISCUSSION

During the last decades, yeasts have been considered as one of the microorganisms which could be used in probiotics (27). This has led microbiologists to seek out the isolation of yeasts from dairy and non-dairy products to explore methods for screening these strains and subsequent evaluations for specific probiotic criteria. In this regards, *S. cerevisiae* var. *boulardi* has been emerged as a well-known probiotic and has been widely applied in industry and health as it has beneficial effects such as improving intestinal conditions (8, 9). Other studies have been reported the probiotic capacity of other yeasts, including *Issatchenkia occidentalis* (6), *K. marxianus* (6, 7) and *Kluveromyces lactis* (7). In the present study, by means of biochemical and molecular methods, we identified strains of *S. cerevisiae* and *K. marxianus* with probiotic potential. Given that identification techniques, which are based on morphological, biochemical and physiological characteristics may result in uncertain classification, these techniques are barely reproducible (28). Therefore, molecular methods are usually considered as more reliable ones. In this study, we confirmed our biochemical-based identification of the selected isolates with DNA sequencing.

Adapting the specific conditions of gastrointestinal tract is a crucial property for considering a microorganism as probiotic (25). Therefore, the isolates were surveyed for tolerance against different temperatures, high concentration of bile salt and acidic pH. Our study demonstrated that both *S. cerevisiae* and *K. marxianus* isolates were able to grow at 37°C and pH=1.5 and withstand to 5% oxbile and pepsin and exhibited the proteolytic activity. This observation emphasizes that these isolates could serve as efficient probiotic candidates. In a previous study, it has been demonstrated the yeasts are also able to grow under low pH (2.0) and 0.6% (w/v) bile salts and survival rates of isolates was higher than 98% (21). Fadda et al. isolated six *Kluyveromyces* strains from artisanal Fiore Sardo cheese and declared that all strains were able to grow in the presence of conjugated bile salts after 72 h of incubation, and showed a percentage survival higher than 95% after exposure to pH 2.0 and in presence of 0.3% (w/v) bile salts. They also reported that all strains survived well under simulated gastric conditions (pH 3.0); *K. marxianus* strains exhibited the best survival rate values (83 to 100%) after contact to artificial duodenum juice (29). In contrast, Diosma et al. (6) obtained a strain of *S. boulardii*, isolated from a commercial probiotic product, which was more sensitive to bile salts than several strains isolated from kefir while 20 isolates grew well even when the concentration was as high as 1 % (w/v).

Foods with supplementary probiotic microorganisms not only need to be safe at the expiration of shelf-life, but must also keep their functional characteristics throughout the same period (30). In this respect, the ability of growth and survival of the probiotic yeast in dairy products during shelf-life and stored period could be monitored. According to the WHO/FAO definition, a standardized probiotic food must contain a minimum of 10^6^ CFU/g active and live organisms at the time of consumption (1). To assure their survival, we have shown that the yeast species *Kluyveromyces* is capable of growth into the yogurt and maintaining cell counts exceeding 10^6^ CFU/ml while the viability of *Saccharomyces* strains in dairy samples was lower than 10^6^ CFU/ml. This is not in agreement with results obtained by Hattingh and Viljoen (26). They showed the mean count of *S. boulardii* in fruit yogurt increased from an initial cell population of 7.7 log10 CFU/ml to 8.1 log10 CFU/ml over the storage period (5°C for 29 days). In addition, Fleet and Mian (31) reported that *S. cerevisiae*, could reach counts as high as 10^8^–10^9^ cells/ml when grown in UHT treated milk. In another study, four dairy-associated yeasts such as *K. marxianus*, associated commonly with yoghurt were isolated. When inoculated into UHT milk, *K. marxianus* populations showed a slight increase from log 7.4 to log 7.7 while *K. marxianus* cell densities remained constant in all the yoghurt products (32).

Changes in the pH during the storage time may be due to the metabolic activity of the probiotic. The decline in pH is accompanied with the increased concentration of organic acids (lactic acid and acetic acid) during storage by probiotic organisms (33). Decreasing the pH of yoghurt around 4.1 to 4.5, in present study, by the identified strains also would inhibit the growth of any pathogenic organisms (33). Proteinases and peptidases constitute the early enzymes in microorganisms responsible for proteolysis in milk proteins as an origin of nitrogen and amino acids (32). In addition, proteolysis and lipolysis have been found to be the main biochemical reactions occurring during ripening of cheese (30). In our study, proteolytic activity was detected in all yeast isolates; therefore, they could result in the better effect on the overall quality of the dairy products.

## CONCLUSION

Our results showed that the *K. marxianus* and *S. cerevisiae* strains, isolated from dairy and non-dairy products, have considerable probiotic features. These isolates were able to grow at 37°C and pH=1.5, and withstand to 5% oxbile and pepsin and exhibited the proteolytic activity. The isolates of *K. marxianus* showed better viability in dairy (yogurt). A further investigation is essential in order to clarify antimicrobial activity of these *K. marxianus* strains and evaluate their capacity to adhere to intestinal epithelium.
